# The Use of Microbial Modifying Therapies to Prevent Psoriasis Exacerbation and Associated Cardiovascular Comorbidity

**DOI:** 10.1007/s10753-023-01915-1

**Published:** 2023-11-13

**Authors:** Eva Reali, Cristiana Caliceti, Antonello Lorenzini, Paola Rizzo

**Affiliations:** 1 Department of Translational Medicine, University of Ferrara, Ferrara, Italy; 2 Department of Biomedical and Neuromotor Sciences, University of Bologna, Bologna, Italy; 3 Laboratory for Technologies of Advanced Therapies (LTTA) Centre, University of Ferrara, Ferrara, Italy; 4 Maria Cecilia Hospital, GVM Care & Research, Cotignola, Ravenna, Italy; 5 Istituto Nazionale Biosistemi e Biostrutture (INBB), Rome, Italy

**Keywords:** psoriasis inflammation, endothelial dysfunction, atherosclerosis, dysbiosis, probiotics

## Abstract

Psoriasis has emerged as a systemic disease characterized by skin and joint manifestations as well as systemic inflammation and cardiovascular comorbidities. Many progresses have been made in the comprehension of the immunological mechanisms involved in the exacerbation of psoriatic plaques, and initial studies have investigated the mechanisms that lead to extracutaneous disease manifestations, including endothelial disfunction and cardiovascular disease. In the past decade, the involvement of gut dysbiosis in the development of pathologies with inflammatory and autoimmune basis has clearly emerged. More recently, a major role for the skin microbiota in establishing the immunological tolerance in early life and as a source of antigens leading to cross-reactive responses towards self-antigens in adult life has also been evidenced. Gut microbiota can indeed be involved in shaping the immune and inflammatory response at systemic level and in fueling inflammation in the cutaneous and vascular compartments. Here, we summarized the microbiota-mediated mechanisms that, in the skin and gut, may promote and modulate local or systemic inflammation involved in psoriatic disease and endothelial dysfunction. We also analyze the emerging strategies for correcting dysbiosis or modulating skin and gut microbiota composition to integrate systemically existing pharmacological therapies for psoriatic disease. The possibility of merging systemic treatment and tailored microbial modifying therapies could increase the efficacy of the current treatments and potentially lower the effect on patient’s life quality.

## INTRODUCTION

Psoriasis is an inflammatory disease of the skin that affects about 2%  of the population. The cutaneous form of the disease is associated with important systemic manifestations and in up to 30% of cases, with psoriatic arthritis (PsA) [1–3].

Regarding the pathogenesis, psoriasis is a complex disease with a strong genetic basis and an autoimmune component. The formation of the psoriatic plaques is based on the interaction between keratinocytes, T cells, dendritic cells, and cells of the microvascular endothelium. This interaction is initially triggered by external events that generate an inflammatory cycle that self-sustains developing around the axis TNFα/IL-23/IL-17 [4, 5].

External triggering events are represented by mechanostressors, drugs, and exposure to UV light that in keratinocytes cause the production of LL-37 antimicrobial peptide and self-DNA release.

The uptake of the complexes LL-37-self-DNA/RNA, by myeloid dendritic cells (mDC) or plasmacytoid DC (pDC), leads to the secretion of TNFα, IL-23, and IL-12 by mDCs and IFNα by pDCs through the stimulation of TLR7/8 and TLR9 [6, 7].

CD11c^+^ inflammatory mDCs are present at higher frequency in psoriatic lesions and express TNFα, IL-23, and iNOS. DCs activated by antigen encounter migrate to lymph nodes draining the cutaneous compartment and can prime naïve T driving their polarization towards Th17/Tc17 phenotype, through the production of IL-1β, IL-6, and IL-23 and towards a Th1/Tc1 phenotype through the production of IL-12 [8–13].

IL-17A produced by Th17/Tc17 cells is a key player in psoriasis pathogenesis; it acts on keratinocytes by inducing the secretion of chemokines and inflammatory molecules that can recruit neutrophils, macrophages, and more T cells to the site of inflammation [14]. Importantly, Th17 cytokines such as IL-22 also directly act on keratinocytes to stimulate proliferation leading to acanthosis and favoring the establishment of a positive feedback loop [15–20].

Psoriasis has also an autoimmune component identified through the detection of T cells reactive to self-antigens. This includes the antimicrobial peptide LL-37, ADAMTSL5 derived from melanocytes, the PLA2G4D lipid antigen, and keratin 17 [21, 22]. CD8^+^ T cells residing in the epidermis and expressing both IFNγ and IL-17 have been indicated as key players in the autoimmune response in psoriasis patients as they are present at the site of disease recurrence [23, 24].

Furthermore, evidence provided in the last years supports a role of T cells recirculating from the skin and specific for self-antigens as one of the missing links between psoriasis and its extracutaneous manifestations [25, 26].

This review is aimed at providing an overview of mechanisms through which alteration in skin and gut microbiome could be involved in the development of psoriasis and the possible association with cardiovascular comorbidities and other systemic diseases. This discussion sets the stage for a critical evaluation of microbial modifying therapeutic approaches to contrast, at least in part, psoriasis exacerbation.

## PSORIASIS AND CARDIOVASCULAR COMORBIDITY

Psoriasis is associated with cardiovascular comorbidities and independently increases cardiovascular risk. It is now considered a systemic inflammatory condition that finally can lead to insulin resistance and endothelial dysfunction linked to cardiovascular disease [27].

The biomarkers of inflammation such C-reactive protein, ESR (erythrocyte sedimentation rate), and P-selectin were found to be increased in the blood of patients with psoriasis and to correlate with disease severity. In addition, imaging techniques showed sites of inflammation in extracutaneous tissues [28–30]. In particular, Mehta and coworkers evidenced vascular inflammation in patients with psoriasis, through F-fluorodeoxyglucose positron emission tomography computed tomography (PET-CT) [31, 32].

Endothelial dysfunction, defined as the inability for arteries to dilate, considered the first step towards formation of atherosclerotic plaques [33], has also been documented in psoriasis patients. Specifically, there is evidence of a correlation between the severity of the disease and the markers of endothelial dysfunction such as asymmetric dimethylarginine (ADMA), reduced levels of circulating endothelial progenitor cells, and integrity of the glycocalyx [34]. Immunological mechanisms shared by psoriasis and atherogenesis may partly explain this phenomenon, and it is increasingly evident that therapies for psoriasis targeting soluble cytokines involved in both psoriasis and atherosclerosis mechanisms can potentially reduce the risk of cardiovascular events [35].

To explain the association between psoriasis and cardiovascular comorbidities, Boehncke and colleagues have introduced the concept of “psoriatic march” that proposes a role for the soluble inflammatory molecules released from the skin to the systemic circulation. Along this line, in psoriasis patients, it has been observed that the cardiovascular risk correlates with the severity of the disease and duration of psoriasis [2, 36–42].

Importantly, studies in mouse models have provided evidence of a causal link between chronic skin inflammation and vascular inflammation [43]. The mouse model K14-IL-17A^ind/+^ overexpressing IL-17A in keratinocytes developed a severe form of psoriasis-like inflammation that was associated with increased CD11b^+^ proinflammatory myeloid cells in the circulation and with increased reactive oxygen species as well as endothelial dysfunction [44]. These findings strongly support the clinical evidence that severity and duration of cutaneous inflammation can also influence vascular inflammation [45].

To this end, it is important to notice that psoriasis patients also have increased number of circulating T cells producing IL-17 and increased levels of IL-17 A in the blood serum compared to healthy controls [46]. Therefore, it is possible that T cells egressing from the skin to reach the systemic circulation could represent a mechanism that links psoriasis with its extracutaneous manifestations.

The analysis of the transcriptome in biopsies from psoriatic skin lesions and atherosclerotic plaque biopsies indeed shows that *TNF*α, *IFN*γ, and IFNγ-induced genes were upregulated to a similar level in psoriasis and atherosclerosis therefore representing putative common pathogenic mechanisms [47]. Conversely, the genes encoding IL-17A and CCL20 were expressed at higher level in psoriatic skin than in atherosclerotic plaques. In atherosclerotic plaques, the level of IL-17A expression associates with neutrophil infiltration suggesting that the axis IL-17A/neutrophils is involved in atherogenesis. Nevertheless, the overall effect of IL-17A in cardiovascular comorbidity associated with psoriasis is still partially unclear [47–49].

## DYSREGULATION OF SKIN MICROBIOME IN PSORIASIS

Skin, the largest organ of the body that provides a physical barrier to injury and microbial insults, harbors abundant and diverse collection of millions of bacteria, fungi, and viruses. Skin microbiome bacteria are mostly from Firmicutes, Actinobacteria, Bacteroidetes, and Proteobacteria phyla [50]. Due to variations in temperature, moisture, and pH value of the skin at different body regions, each site provides a unique colonization environment and, therefore, favors the survival of some bacteria over others.

The commensal bacteria present in the skin under normal conditions favor the maintenance the immunological homeostasis through mechanisms that include induction of tolerogenic dendritic cells in early life and priming of regulatory T cells protecting from immune responses towards commensal-derived antigens. Emerging evidence indicates that skin dendritic cells can present antigens from skin microbiota and that this mechanism, in neonatal life, is essential to correctly develop tolerance to commensals [51, 52]. For this reason, a decrease of commensal bacteria and the increase of non-commensal species could alter the skin immune homeostasis, favoring the generation of inflammatory responses and leading to impaired barrier functions [53].

Analysis of the skin microbiome in psoriatic plaques and normal skin of patients with psoriasis as well as in the skin of healthy subjects indicated, as a major variation, a decrease in microbial diversity in psoriatic plaques. Specifically, there was a variation in the relative abundance of Firmicutes, Actinobacteria, and Proteobacteria with a prevalence of Firmicutes in psoriatic skin lesions and a significantly lower level of Actinobacteria compared to healthy and non-lesional skin. Other studies report a decreased representation of *Cutibacterium*, *Burkholderia* spp., and *Lactobacilli* in psoriatic skin. By contrast, the abundance of *Corynebacterium* was increased in lesional skin and associated with the severity of inflammation [54, 55]. An increased abundance of *Streptococcus* spp. in psoriatic skin has also been reported whereas *Staphylococcus epidermidis* was more common in normal skin [53, 56]. Streptococcal infections have been known for a long time for its association with guttate psoriasis, and only more recently, it has been associated also with the exacerbation of plaque psoriasis (40, 45, 46). The evidence that the streptococcal-derived superantigens can activate a subset of T cells in a peptide-antigen-independent manner could provide an explanation of this phenomenon. Importantly, in pathological conditions, keratinocytes express HLA-DR molecules and can present streptococcus-derived superantigens to T cells [57–59]. Noticeably, M protein of *Streptococcus pyogenes*, which colonizes psoriatic lesions, shows molecular mimicry with keratin 17 [60–62] and has been suggested to have a role in the activation of T cells cross-reacting with keratin-derived self-antigens as discussed in paragraph 5.

## THE GUT-SKIN AXIS IN PSORIATIC DISEASE

Besides the evidence of the direct effect of skin microbiome on skin diseases, the intestinal microbiota also communicates with the skin providing evidence of a gut-skin axis [63].

**Fig. 1:**
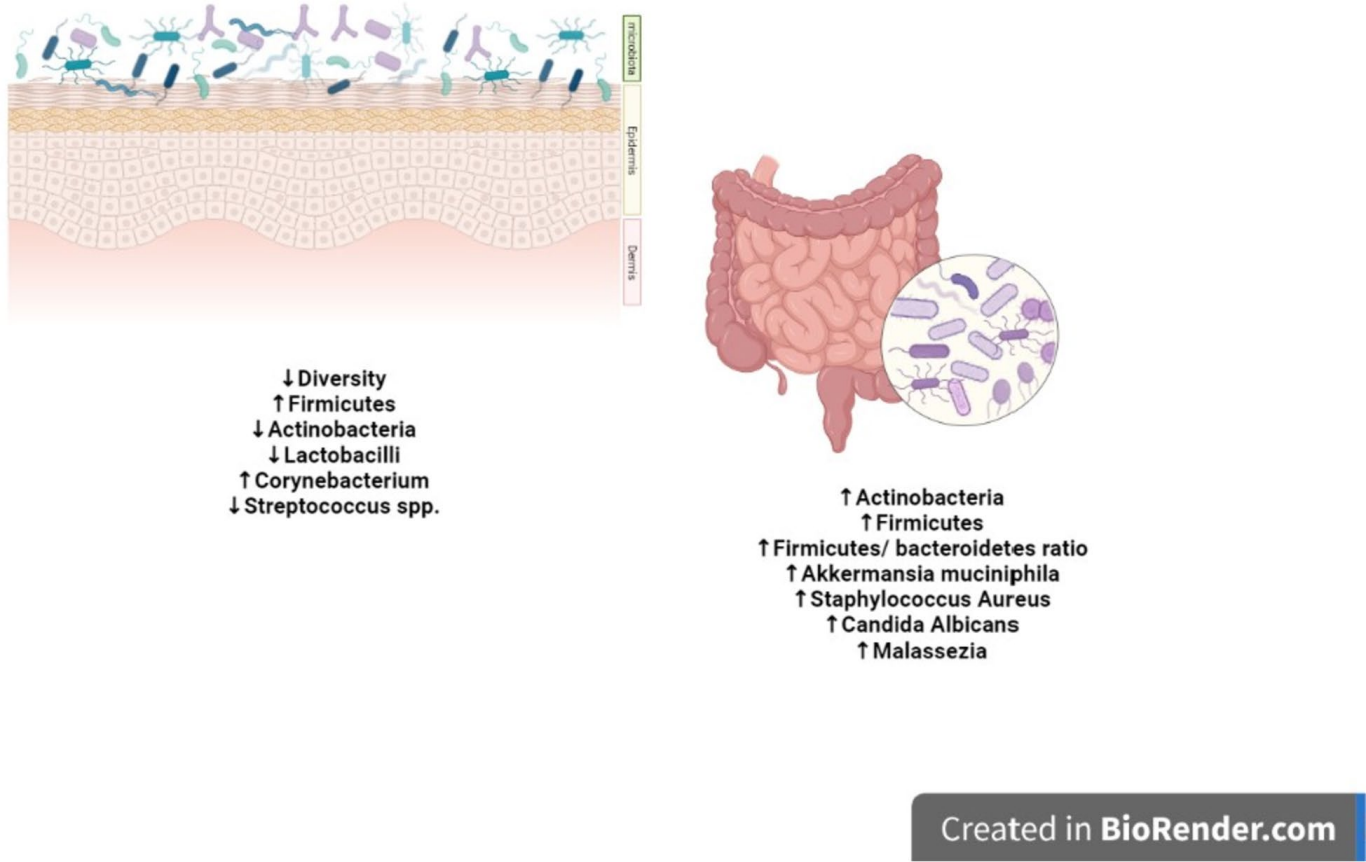
Skin and gut dysbiosis in psoriasis and associated comorbidities. Original figure created by Biorender.com.

Human intestinal microbiota comprises bacteria mainly belonging to six phyla: Bacteroides, Actinobacteria, Fusobacteria, Firmicutes, Verrucomicrobia, and Proteobacteria, fungi, viruses, protozoa, and archaea [64]. In psoriasis patients, the intestinal microbiome has however a modified pattern that is characteristic of impaired intestinal barrier function and shares features with other intestinal inflammatory pathologies, including inflammatory bowel disease. This includes increased abundance of Actinobacteria and Firmicutes and increased Firmicutes-to-Bacteroidetes ratio (F/B ratio). Moreover, there is evidence indicating that the exacerbation of psoriasis is strongly associated with increased abundance of *Staphylococcus aureus*, *Candida albicans*, and *Malassezia* [55, 65] (Fig. 1).

A recent study in the keratinocyte-specific caspase-1 transgenic (Kcasp1.Tg) mouse model of skin inflammation [66] showed that the fecal microbiome was characterized by abundance of *Staphylococcus aureus* and *Streptococcus danieliae*. Accordingly, when wild-type mice treated with antibiotics were administered orally with *Staphylococcus aureus* and *Streptococcus danieliae* before the induction of psoriasis-like inflammation with imiquimod, there was an increase in the severity of skin inflammation. This evidence definitively points towards the existence of a bidirectional axis in which skin inflammation can modify the gut microbiome and modifying the gut microbiome can favor skin inflammation.

A study in patients with psoriasis has reported an impairment in the intestinal barrier [67]. The assessment was based on the quantification of the plasma level of intestinal fatty acid-binding protein (FABP) and claudin-3 component of the tight junction that was used as an indicator of damage to the enterocytes. In these patients, the increase of the markers of impaired barrier function was associated with a higher disease activity and increased blood biomarkers of systemic inflammation including C-reactive protein and neutrophil-to-lymphocyte ratio. The marker of bacterial translocation trimethylamine N-oxide (TMAO), which is a gut microbiota-associated metabolite, was markedly increased in the plasma of psoriasis patients with altered gut integrity [67]. From this evidence, it emerges that, in patients with psoriatic disease, intestinal dysbiosis is associated with an intestinal barrier impairment and translocation of bacteria that therefore acquire access to the immune cell compartment. The phenomenon is called “leaky gut” and has been associated with several extraintestinal autoimmune diseases.

Differences in microbiota composition compared to healthy subjects have been shown by sequencing-based approaches in autoimmune diseases including rheumatoid arthritis, multiple sclerosis, type 1 diabetes, and systemic lupus erythematosus [68].

As regards rheumatoid arthritis (RA), a higher fecal level of *Clostridium perfringens* in patients was reported already in the’60, and recently, next-generation sequencing studies highlighted increased fecal levels of *Prevotella copri*, in RA patients [69, 70].

## IMPORTANCE OF CROSS-REACTIVITY IN THE PREDISPOSITION TOWARDS PSORIASIS AND OTHER AUTOIMMUNE PATHOLOGIES

Among the mechanisms that could underlie the development of autoimmune diseases, there is molecular mimicry, based on structural similarities between proteins derived from infectious agents or commensal bacteria and proteins from the host. This phenomenon could cause the activation of T and B cells cross-reacting with self-proteins. It is increasingly evident that commensals and pathogen-derived antigens can induce cross-reactive T cells. For instance, in a subgroup of patients with rheumatoid arthritis, the HLA-DR-presented peptide from a 27-kd protein (Pc-p27) derived from *Prevotella copri* was shown to activate T cells and B cells. Two other autoantigens were identified in rheumatoid arthritis that have T cell epitopes similar to peptide antigens derived from *Prevotella* and *Parabacteroides*.

In the context of psoriasis, it is of note that the CD8^+^ T cells specific for streptococcal M protein cross-recognizing a keratin 17-derived self-peptide have been identified in the circulation of psoriasis patients pointing towards a role for cross-reactive bacterial antigens in the disease pathogenesis. To this end, it has recently been reported that CD8^+^ T cells with a skin-primed phenotype are clonally expanded in the circulation of both a mouse model of autoimmune psoriasis and in patients with psoriatic arthritis [71]. Together, this evidence underlines the importance of preventing the generation of T cell responses to commensals that could cross-react with self-antigens and favor the spreading of inflammatory responses at distant organs.

## THE INTESTINAL AND SKIN DYSBIOSIS IN PSORIASIS AND ASSOCIATED COMORBIDITIES

Intestinal dysbiosis could have a major impact on the development of comorbidities associated with psoriasis.

**Fig. 2:**
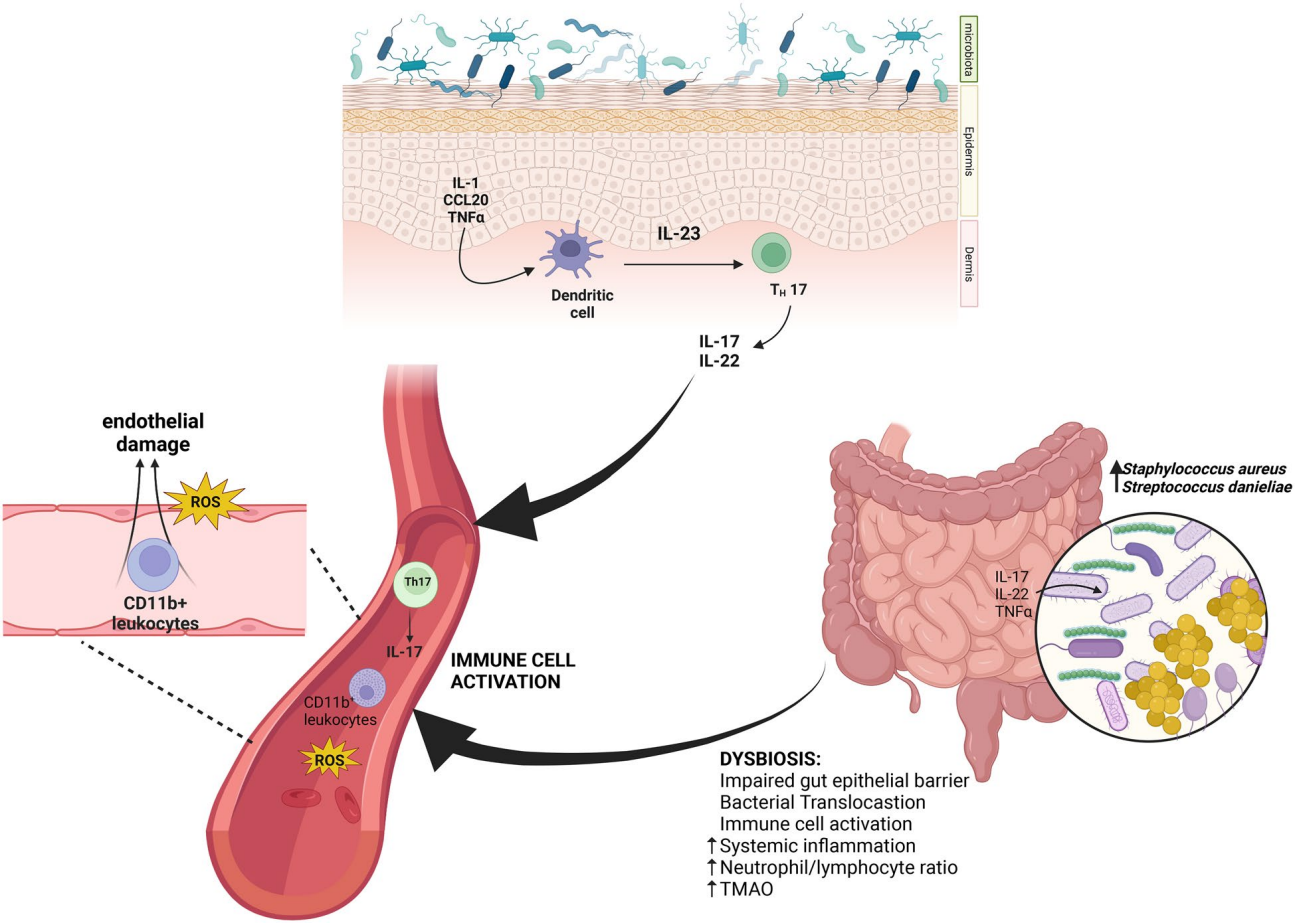
Intestinal dysbiosis and psoriasis and cardiovascular comorbidities: the gut-skin and gut-artery axes. Original figure created by Biorender.com.

High Firmicutes*/*Bacteroides (F/B) ratio has been found in psoriasis and in other diseases associated with systemic inflammation [64, 72, 73]. In healthy subjects, it has been observed that Firmicutes/Bacteroides ratio in the gut was correlated with augmented plasma level of trimethylamine-N-oxide (TMAO), a metabolite with a proatherogenic potential produced by bacteria [74]. TMAO influences the metabolism of cholesterol and activates macrophages and is therefore linked to an increased risk of cardiovascular events [75] (Fig. 2). Clinical trials and observational studies have reported gut dysbiosis in psoriatic patients characterized by decreased abundance of *Akkermansia muciniphila* and increased *C. citroniae* [55]. In another study, higher diversity of intestinal microbiota was reported in patients with psoriasis compared with healthy subjects [76]. Psoriatic microbiome in this case showed an increased abundance of *Faecalibacterium*, *Akkermansia*, and *Ruminococcus*, while levels of *Bacteroides* were found to be decreased. Conversely, in another study, a decrease in bacterial diversity and *Akkermansia* and *Ruminococcus* levels were observed in psoriatic patients [77, 78]. These differences might have arisen due to variation in the study design.

## PROBIOTICS IN PSORIASIS

According to the definition of Joint FAO/WHO Expert Committee on Food Additives of 2002, probiotics are microorganisms that administered in adequate amounts provide health benefits to the host [79]. They can exert their activity through different mechanisms: (i) they can prevent gut colonization by harmful bacteria; (ii) they stimulate the function of the mucosal barrier; (iii) they regulate immune cell function, in particular Tregs to prevent excessive response inflammatory signals and commensals; and (iv) they can release metabolites with anti-inflammatory activity.

Among probiotics, the lactic acid bacteria (LAB) family, mainly found in dairy food, substantially contributes to maintain the homeostasis of gut microbiota through the fermentation of food fibers.

The cross-talk between intestinal microbiota and immune cells enables a balanced intestinal homeostasis in healthy individuals. However, an alteration due to the aged gut or diseases causes changes in gastrointestinal microflora equilibrium, resulting in several chronic diseases. The first explanation of the beneficial effects of probiotics was provided by the “hygiene hypothesis,” suggesting that a lack of exposure to microbial stimuli early in childhood was the major factor behind allergic reactions. The interaction of probiotics and intestinal microflora with the gut-associated lymphoid tissues (GALT) indeed favors the induction of oral tolerance and mucosal immunity [63, 80].

Interestingly, in the mouse model of imiquimod-induced psoriasis-like inflammation, early exposure to oral antibiotics leads to exacerbation of a more severe form of psoriasis in the adult life. This provides a pivotal evidence of the role of gut-skin axis and the importance of its alteration in predisposing to inflammatory skin disease [81].

The intestinal microbiota and ingested probiotics can sustain the establishment of immune tolerance by activating tolerogenic DCs in the gut that drive Treg cell differentiation. This occurred through a decrease of the expression of CD80, CD83, and CD86 costimulatory molecules on DCs and expression of polarizing cytokines such as IL-12 together with an increase of indoleamine 2,3-dioxygenase (IDO) and IL-10. These changes promote unresponsiveness of the immune cells to self-antigens that is critical for homeostatic maintenance [82].

The mechanisms through which probiotics promote immune tolerance and impair unwanted proinflammatory responses is largely mediated by the production of SCFAs following fermentation of dietary fibers.

SCFAs are saturated fatty acids comprising formate (C1), acetate (C2), propionate (C3), butyrate (C4), and valerate (C5). The beneficial activity of SCFAs is due to the positive effect on regulatory T cell differentiation and on the production of IL-10. The activity of these molecules is mainly exerted locally; however, it has been shown that through the peripheral blood they can exert their effect also at the systemic level in tissues other than the intestine [83].

As a mechanism of action, the intestinal absorption of SCFAs is favored by substrate transporters like MCT1 and SMCT1 that regulate the intracellular concentration of butyrate in colonic epithelial cells. Moreover, SCFAs can activate the cell surface G protein-coupled receptor (GPCR) signaling cascades thus controlling immune cell functions [84]. SCFAs also control energy consumption by modulating glucose and lipid metabolism, maintaining the integrity of the mucosal barrier [85].

As a mechanism of action for inhibiting pathogenic bacteria translocating from the gut, LAB compete with enteric pathogenic bacteria for binding to mucin sites on the surface of epithelial cells. The effects of lactic acid bacteria on the cells of the immune system have supported their use in several preclinical and clinical studies in allergic and autoimmune disorders. Moreover, evidence was provided that heat-killed *Lactobacillus sakei* proBio65 administered orally could inhibit the release of histamine and β-hexosaminidase mediated by immunoglobulin E in NC/Nga mice, supporting a potential inhibitory effect on atopic dermatitis-like skin lesion [86, 87]. *Lactobacillus casei* upon oral administration was reported to reduce antigen-specific skin inflammation [88, 89]. Rather et al. have described that skin application of *Lactobacillus sakei* proBio65 reduced psoriasiform inflammation as well as the expression of IL-17, IL-19, and IL-23 [90].

Oral administration of *Lactobacillus pentosus* GMNL-77 and *Bifidobacterium infanti*s reduced the clinical signs of psoriasiform inflammation in the mouse model as well as in humans [76, 91]. Because of the increasing amount of evidence in recent years, considerable focus has been directed towards the anti-inflammatory properties of probiotics aimed at restoring the functional protective ecosystem. In particular, *L. rhamnosus* and *L. delbrueckii* are known for their induction of tolerogenic DCs and their effect on the stimulation of Treg cells [92–94] (Table 1).

**Table 1:** Summary of the Models and Results of Preclinical Studies and Clinical Trials

**Study**	**Probiotics/prebiotics/MMTs**	**Model and administration route**	**Outcome**	**Ref**
***Skin inflammation***				
**Preclinical mouse**	*Lactobacillus sakei* proBio65 resistant to gastric acidity	Mouse model of atopic dermatitis by allergen DNCB Oral administration *L. sakei* proBio65	Increased recovery compared to control Decreased serum level of (Ig) E and (IL)-4	[86]
**Preclinical mouse**	Fermented milk containing *L.* *casei* or *L.* *casei*	Mouse model of CHS 2,4-DNFB mediated by CD8^+^ CTL and controlled by CD4^+^ Tregs Oral administration daily	Decreased skin inflammation by inhibition of the priming/expansion of hapten-specific IFN-γ-producing CD8^+^ effector T cells	[88]
**Preclinical mouse**	Lactobacillus sakei proBio65 isolated from kimchi	NC/Nga mouse model artificial induction of atopic dermatitis Oral administration	Decreased serum level of IL-6 and IL-4 and CTACK (CCL27), IgE Improved skin conditions	[87]
**Preclinical mouse**	*L. casei* (DN-114 001)	Mouse model of epicutaneous (CHS) sensitization with DNFB (DTH) to ovalbumin in CFA+ OVA challenge Oral administration 2 weeks before sensitization	Reduced CHS Decreased skin DTH response Reduced severity of DTH responses mediated by Ag-specific CD4+ or CD8+ T cells Increased frequency of skin resident CD4+ CD25+ FoxP3+ Treg	[89]
***Psoriasis-like inflammation (IMQ)***				
**Preclinical mouse**	*Lactobacillus sakei* proBio65 ethanol extract (SEL001)	Mouse model of imiquimod (IMQ)-induced psoriasis-like skin inflammation Topical application 1 h before application of IMQ Control with standard drug clobetasol	Inhibition of the severity of skin inflammation by both clobetasol and SEL001 Reduced mRNA expression of proinflammatory cytokines, including IL-19, IL-17A, and IL-23	[90]
**Preclinical mouse**	Sodium butyrate (SB)	Mouse model of imiquimod-induced psoriasis-like skin inflammation model Topical application of SB	SB reduced imiquimod-induced inflammation, downregulated IL-17 and induced IL-10 and FOXP3 transcripts. The effect of SB was ascribed to Tregs. Reduced suppressive activity of Tregs from psoriasis patients was restored upon *in vitro* treatment with SB.	[115]
***Autoimmune disease***				
**Preclinical mouse**	Short-chain fatty acid, propionic acid (PA)	Mouse model of EAE in high-fat diet PA in water Treated either with water or PA Explorative study in obese and non-obese MS patients	PA treatment prevented disease-enhancing effect of HFD by inhibiting Th17-mediated inflammatory processes in gut and spleen. Small group of MS patients’ PA intake could restore the Treg-Th17 homeostasis.	[95]
***Cardiovascular disease***				
**Preclinical rat**	*C. butyricum*	Spontaneously hypertensive rat (SHR)-induced hypertension 6 weeks oral administration *C. butyricum* and captopril	*C. butyricum* modulated SHR-induced dysbiosis and significantly reduced systolic blood pressure.	[98]
	*Lactiplantibacillus plantarum*	2 parallel-armed double-blind placebo-controlled interventions (*n* = 136 and *n* = 104), daily test yoghurt (Inducia) or placebo yoghurt BMI, blood pressure, plasma glucose, cholesterol, high-sensitivity C-reactive protein (hs-CRP), oxidative stress, and immunological markers were measured	Significant reduction of total cholesterol (LDL-c) and non-high-density cholesterol A difference was also found between placebo and test yoghurt groups (*P* = 0.042) in LDL-c with normal BMI. Blood glucose reduction (*P* = 0.01) antioxidative effect in overweight volunteers of the test yoghurt group	[99]
**Preclinical rat**	*Limosilactobacillus fermentum 139*, *L. fermentum 263*, and *L. fermentum 296* Mixed formulation	Female rats fed a high-fat diet (HFD) twice a day for 4 weeks Cardiometabolic parameters, inflammatory markers, short-chain fatty acid (SCFA) fecal contents, and oxidative stress in colon, liver, heart, and kidney tissues	Increased acetate and succinate fecal contents Reduced hyperlipidemia and hyperglycemia in rats fed with HFD Decreased low-grade inflammation Improved antioxidant capacity along the gut, liver, heart, and kidney tissues	[101]
***Clinical trials and case studies***				
**Clinical trial**	*B. infantis* 3562	3 separate randomized, double-blind, placebo-controlled interventions Patients with ulcerative colitis (UC) (*n* = 22), chronic fatigue syndrome (CFS) (*n* = 48), and psoriasis (*n* = 26) Oral administration, for 6‒8 weeks Inflammatory biomarker and plasma cytokine levels	Reduced plasma CRP levels in all 3 inflammatory disorders compared with placebo Reduced plasma TNF-α in CFS and psoriasis	[91]
**Clinical trial**	*Bifidobacterium longum* CECT 7347, *B. lactis* CECT 8145, and *Lactobacillus rhamnosus* CECT 8361 Total 1 × 10^9^ CFU per capsule	12-week randomized, double-blind, placebo-controlled trial Oral administration	12-week follow-up, reduction in PASI of at least 75% (*P* < 0.05) and PGA 6 months after the end of the study showed a lower risk of relapse	[96]
**Case study**	FMT twice *via* both upper endoscopy and colonoscopy with a 5-week interval	36 years old, diagnosed as severe plaque psoriasis and irritable bowel syndrome (IBS) Body surface area (BSA), PASI, dermatology life quality index (DLQI), histological examination, intestinal symptoms, adverse reactions	After the second FMT treatment for 5 weeks, all parameters were improved greatly compared with those before treatment.	[104]
**Clinical trials**	FMT	Double-blind, parallel-group, placebo-controlled, superiority trial, FMT or sham transplantation into the duodenum 31 patients underwent randomization (15 FMT); 30 completed the 26-week clinical evaluation.	No serious adverse events were observed. Treatment failure occurred more frequently in the FMT group than in the sham group.	[106]

*DNCB* 1-hypersensitivitychloro-2,4-dinitrobenzene, *IMQ* imiquimod, *CHS* contact hypersensitivity, *DNFB* 1-fluoro-2,4-dinitrobenzene, *DTH* delayed-type hypersensitivity, *CFA* complete Freund’s adjuvant, *OVA* ovalbumin, *PA* propionic acid, *EAE* experimental autoimmune encephalomyelitis, *PASI* psoriasis area and severity index, *PGA* physician global assessment, *FMT* fecal microbiota transplantation

## CROSS-TALK BETWEEN PROBIOTICS AND INTESTINAL MICROBIOTA IN REGULATING INTESTINAL EPITHELIUM AND SYSTEMIC IMMUNE RESPONSES

Dietary fibers as well as pre- and probiotics are available tools to increase peripheral SCFA concentrations (50). Propionate was shown to be beneficial in the case of high-fat diet and to reduce the cardiometabolic risk in patients with psoriasis [95].

Two clinical studies have been reported so far on probiotic administration in patients with plaque psoriasis. First, a study conducted on 22 patients with ulcerative colitis, 48 patients with chronic fatigue syndrome, 22 patients with chronic plaque psoriasis, and 35 healthy control individuals showed that administration of 10^10^ colony-forming units of viable *Bifidobacterium infantis* per day for 6–8 weeks significantly decreases plasma levels of CRP and TNF-α. Importantly, it was also shown that the production of TNF-α and IL-6 by PBMCs from healthy individuals upon ex vivo stimulation with LPS was lower in group receiving *B. infantis 35,624*-treated groups compared to the group receiving placebo indicating a modulation of the immune response at the systemic level [91].

Secondly, a study in 90 patients who experienced the administration for a total 12 weeks of three probiotic strains *Bifidobacterium longum* CECT 7347, *B. lactis* CECT 8145, and *Lactobacillus rhamnosus* CECT 8361 with a total of 1 × 10^9^ CFU daily showed a decrease in the PASI score compared to patients receiving placebo. Moreover, a follow-up of 6 months evidenced lower risk of psoriasis relapse in patients who had received probiotics [96] (Table 1).

As regards atherosclerosis and CVD risk, probiotics have been reported to be beneficial in a preventive setting by numerous studies [97–101]. Among the mechanisms responsible for this phenomenon, the bile salt hydrolases produced by the bacteria can increase the conversion of cholesterol to bile acids increasing fecal excretion of bile acids thus favoring the decrease of cholesterol level in the blood l [102].

In particular, clinical studies evidenced that *Lactobacillus* probiotics as well as candidate next-generation probiotics such as *Akkermansia muciniphila* and *Faecalibacterium prausnitzii* are efficient in reducing cholesterol levels [102].

Obesity is also linked to CVDs, and clinical trials have reported that dietary supplementation with both probiotic and prebiotic can counteract this condition [103] (Table 1). In the light of this evidence, it is encouraged to design specific clinical studies, to support the use of probiotics in the prevention and treatment of CVDs.

## OTHER APPROACHES OF MICROBIOTA-TARGETED THERAPY FOR PREVENTION AND TREATMENT OF PSORIATIC DISEASE

The modulation of gut microbiota through the use of probiotics, antibiotics, and fecal microbiota transplantation (FMT) has been employed, with the aim to reverse the established microbial dysbiosis [80] thus treating or preventing various diseases associated with gut dysbiosis.

Fecal microbial transplantation for instance has been investigated as a therapeutic tool in a patient with severe plaque psoriasis and inflammatory bowel disease, who underwent fecal microbiota transplantation twice with an interval of 5 weeks through both upper endoscopy and colonoscopy. In this case, the clinical signs of both psoriasis and inflammatory bowel disease improved, and no considerable adverse effects of intervention were reported [104]. In another study, fecal microbiota from psoriatic patients and healthy individuals were transplanted in mice with psoriasis-like inflammation to compare disease recovery and cytokines levels. Mice transplanted with microbiota from psoriatic patients showed delayed recovery of psoriasis-like inflammation and delayed decrease of IL-17A than mice receiving fecal microbiota from healthy subjects or untreated control mice [105].

Recently, an attempt has also been made aimed at correcting the gut microbiome with the purpose to affect the development and severity of psoriatic arthritis in psoriasis patients by FMT in a cohort of 31 patients. The attempt however failed, and the 15 patients undergoing FMT did not show a better outcome than sham [106] (Table 1).

## NEXT-GENERATION PROBIOTICS AS PUTATIVE PREVENTION AND TREATMENT TOOLS FOR PSORIASIS AND ASSOCIATED CARDIOVASCULAR COMORBIDITIES

Next-generation sequencing (NGS) has enabled a rapid expansion in the range of microorganisms known to have potential benefits for host health. The new microorganisms identified by NGS technology are now called next-generation probiotics (NGPs) as well as live biotherapeutic products (LBPs) [107]. NGPs are defined as “live commensal microorganisms, identified upon comparative microbiota analyses, that when administered in adequate amounts, confer a host health benefit.” This category includes bacteria of the genera *Akkermansia*, *Bacteroides*, and *Faecalibacterium*.

According to the guidelines from the Food and Drug Administration, NGPs are “active biological agents” (i) containing live organisms, such as bacteria; (ii) are applicable for prevention, treatment, or cure of a disease or condition in human beings; and (iii) are not a vaccine. Before entering the market, these NGPs need to be assessed in clinical trials (from phases I to IV) and require approval by the regulatory authorities.

This new type of studies necessary for NGP approval should publish the genomic sequence and evaluate the properties, the profile of antibiotic resistance, and the safety and toxicological profile to fulfill the novel food regulations. Functions of individual strains may be different for specific diseases.

Based on the literature in the field, it is possible to envisage that targeted designed probiotic formulation can be used to specifically interfere with the progression of the psoriatic disease, with the associated psoriatic arthritis, chronic inflammation, and cardiovascular disease.

In people with CVDs, lower levels of bacteria producing butyrate and *Roseburia* have been reported, and in a mouse model, feeding with *Roseburia* together with a high-fiber diet reduced atherosclerosis development [108].

A study in a human cohort showed that the intake of alcohol was associated with lower abundance of *Roseburia*, and in a mouse model of alcohol-related liver disease, it was shown that oral administration of *Roseburia intestinalis* could restore the integrity of the intestinal barrier. Nevertheless, it has also been reported that *Roseburia intestinalis* can exacerbate antiphospholipid syndrome in genetically individuals and susceptible mice through a mechanism that involves T and B cell epitope sharing [109].

Therefore, to establish the precise effect of NGPs on the heath of the patients, complex screening processes and experiments to clarify the underlying mechanisms will be required. To this end, the US FDA has started a program to regulate the use of this promising category of probiotics.

Among the major candidates as NGP to be used in the combinatorial treatment of psoriasis patients, *Akkermansia muciniphila* should be mentioned, as it may represent a key node for psoriasis progression but also for inflammatory bowel disease and obesity [110]. Evidence has been provided showing that *A. muciniphila* is negatively correlated with cardiometabolic conditions and low-grade inflammation; therefore, conceivably, its administration could positively correlate with an improvement of psoriasis course and decreased comorbidity development [111]. Preliminary studies in humans indicated the safety of *A. muciniphila* administered orally; nevertheless, further clinical trials are necessary to finally support this evidence.

*Faecalibacterium prausnitzii*, a member of the Firmicutes phylum, can also participate in the maintenance of the gut homeostasis by producing butyrate [110].

In patients with psoriasis and psoriatic arthritis, Scher et al. and Eppinga et al. reported a decline in *F. prausnitzii* [77, 78, 112]. It is therefore conceivable that correcting this decline could be beneficial for patients’ conditions.

The main mechanism through which NGPs may control the development of cardiovascular illness is the production of SCFAs. Among the mechanisms proposed to explain this effect is the reduction of cholesterol level by (i) decreasing the expression of genes involved in the cholesterol synthesis, (ii) increasing the expression of cytochrome P450 monooxygenase, which accelerates the transformation of cholesterol to bile acids, and (iii) activation of G protein-coupled receptor 41 (GPR41) in adipocytes to produce leptin, which further suppresses the expression of the master regulator of sterol and fatty acid synthesis SREBP2.

A deep understating and complete clinical trial addressing these points could optimize the use of the NGPs in implementing the current therapeutic and preventive tools for the treatment of patients with psoriatic disease. As a mechanism of action, NGP can alter the ecosystem thus correcting dysbiosis and colonization by bacterial strains that favors the generation of pathogen-specific T cells. These cells through molecular mimicry could give rise to cross-reactive T cell responses towards self-antigens that can favor autoimmunity associated with dysbiosis. In addition, NGPs can promote a tolerogenic phenotype in dendritic cell through the production of SCFAs, decrease CD80 CD86 expression, and increase in the generation of Tregs. NGPs could also decrease in the inflammasome activation and the downstream inflammatory cascade that could promote psoriasis exacerbation and increase the barrier integrity and prevention of the leaky gut phenomenon that favors systemic inflammation. Finally, they decrease TMAO and the related cardiovascular risk.

## CHALLENGES FOR THE THERAPEUTIC USE OF MICROBIAL MODIFYING THERAPIES AND CONCLUSIONS

Because of limitations in the use of probiotics due to their decreased viability during the transition to the gut and their safety, recently, new formulations have been developed with the attempt to overcome these limitations and improve the specificity of the intervention. These include prebiotics, postbiotics, and symbiotics.

Prebiotics do not contain microorganisms, and they are resistant to the actions of stomach acid; therefore, they can reach the intestine unaltered and exert their activity. To be considered prebiotics, food components need to have a known chemical structure, provide a substrate for beneficial bacteria, and stimulate the growth of the desired groups of bacteria [113]. Experiments performed in HaCaT human keratinocyte cell line treated with sodium butyrate and an inhibitor of epidermal growth factor receptor showed enhanced keratinocyte differentiation suggesting this combination as a potential tool in the treatment of hyperproliferative skin diseases including psoriasis [114]. In a psoriasis mouse model, cutaneous application of sodium butyrate increased IL-10 and FOXP3 expression in T cells and reduced inflammation [115]. No clinical studies have been completed in psoriasis patients; however, the effect of a lactic acid-based skin treatment is undergoing evaluation in an exploratory study on plaque psoriasis (NCT05078567) [53].

The term postbiotic refers to substances derived from the processing of microorganisms. Postbiotics have to be prepared through a precise and reproducible technological process of biomass production and inactivation [116]. Postbiotics include bioactive compounds generated in matrix during fermentation as well as by heat-killed bacterial strains (*Akkermansia muciniphila* ATCC BAA-835) [116, 117]. Among postbiotics, we have recently reported that a fermentation product of *Vaccinium floribundum* berries with *Lactiplantibacillus plantarum* has an antioxidant effect on human endothelial cells (HUVECs) and immunomodulatory properties in a macrophage cell line [118].

Probiotics can also be combined with prebiotic and these combinations are called symbiotics [119]. In their mechanism of action, probiotics, prebiotics, and symbiotics can decrease the level of cholesterol by increasing the synthesis of bile salts and bile acid deconjugation thus having the potential to provide protection from CVD development.
